# Genome-wide association study of wheat chlorophyll dynamics under drought and irrigation using multispectral UAV phenotyping

**DOI:** 10.3389/fpls.2025.1607862

**Published:** 2025-09-04

**Authors:** Yukun Cheng, Wanlong He, Fuxing Zheng, Feifei Zhang, Bin Bai, Na Sun, Wei Wang, Hongwei Geng

**Affiliations:** ^1^ College of Agronomy, Xinjiang Agricultural University/Engineering Research Center for High-quality Special Wheat Crops, Xinjiang Agricultural University/Innovation Team for Xinjiang Wheat Industry System, Urumqi, China; ^2^ Wheat Research Institute, Gansu Academy of Agricultural Sciences, Lanzhou, China; ^3^ Yili Institute of Agricultural Science, Yining, Xinjiang, China; ^4^ Department of Computer Science and Information Engineering, Anyang Institute of Technology, Anyang, China

**Keywords:** wheat, UAV, drought, genome-wide association analysis, chlorophyll

## Abstract

High-throughput phenotypic analysis using multispectral unmanned aerial vehicle (UAV) technology is a critical approach for enhancing the efficiency and accuracy of gene mining. This study aimed to evaluate the feasibility of UAV-based remote sensing techniques in predicting chlorophyll content and conducting genome-wide association studies (GWAS) for winter wheat under both normal and drought stress conditions. The study was conducted in the fall of 2019 at the Zepu and Manas experimental bases using winter wheat. Chlorophyll content was measured manually during the heading, flowering, and grain filling stages and compared with data obtained via UAV-mounted multispectral sensors. A predictive model for chlorophyll content was developed using UAV data and validated against manual measurements. The predicted and measured chlorophyll values were then integrated into a GWAS to identify loci associated with chlorophyll content.Chlorophyll content values differed across growth stages, with both measured and predicted values increasing from the heading to grain filling stages. Under normal conditions, manual measurements ranged from 43.96 to 65.85, while UAV-predicted values ranged from 47.59 to 62.29. Under drought conditions, manual measurements ranged from 45.00 to 66.33, and UAV-predicted values ranged from 47.83 to 65.89. Correlation coefficients between measured and predicted values were high under normal conditions (0.90–0.93 during heading, 0.91–0.92 during flowering, and 0.88–0.90 during filling) and moderate to high under drought stress (0.57–0.70, 0.89–0.91, and 0.94–0.96, respectively). A neural network model demonstrated high accuracy in predicting chlorophyll content. GWAS revealed 308 loci associated with chlorophyll content, with UAV-predicted data identifying 206 loci across 21 chromosomes, explaining 7.58%–19.58% of the phenotypic variation. Measured values identified 102 loci across 21 chromosomes, accounting for 9.31%–15.83% of the variation. Eighteen overlapping loci were detected on chromosomes 1A, 1B, 2B, 3B, 4B, 5A, 5B, 5D, 6B, 6D, 7A, and 7B. This study confirms the reliability of UAV-based multispectral data for chlorophyll content inversion and GWAS. Site-specific differences in prediction quality were observed, with site P showing stronger correlations and higher prediction accuracy. Analysis of loci identified 21 candidate genes potentially related to chlorophyll content, including those encoding chlorophyll a/b-binding proteins, aquaporins, and chlorophyll kinases. These findings demonstrate the potential of UAV technology for high-throughput, efficient, and accurate phenotyping, facilitating better understanding of the genetic mechanisms underlying chlorophyll content variation.

## 1 Introduction

The chlorophyll content affects photosynthetic efficiency and crop growth. One approach to improving wheat production is the timely acquisition and analysis of the chlorophyll content of plants during the pre-production stage (Peng and Gitelson, 2011; Li et al., 2021; Roy et al., 2021). Currently, wheat chlorophyll contents are primarily determined using a chlorophyll meter (Shu et al., 2021) and spectrophotometer (Kyslychenko et al., 2019). Both methods are expensive, inefficient, have large random errors, and are inappropriate for collecting information for large-scale phenotyping. The continual maturation of remote sensing technologies has enabled the extensive use of drones for high-throughput phenotyping relevant to agricultural production and research (Wang et al., 2021). Spectroscopic information is gathered by drones and retrieved to determine the chlorophyll content. Determining the chlorophyll content using an inversion model has certain advantages (e.g., high efficiency, low cost, considerable range of field information, and low learning cost) (Zhang et al., 2021).

Various sensors have been used for the inverse modeling of plant chlorophyll contents. For example, Qiao et al. (2019) used unmanned aerial vehicles (UAVs) to obtain maize R, G, and B bands as well as the back propagation (BP) neural network for an inversion model-based analysis of the chlorophyll content. The coefficient of determination (*R^2^*) value of the model was 0.72, with a root mean squared error (RMSE) of 4.47. Wei et al. (2020) used UAVs to acquire various spectral data for wheat and selected 16 spectral vegetation indices using a stepwise regression method to improve the monitoring of wheat chlorophyll contents (*R^2^* = 0.81 and RMSE = 1.68). Additionally, Zhou et al. (2020) used wheat canopy spectral data obtained from drones for the inversion model-based estimation of wheat chlorophyll contents using a stepwise regression method (*R^2^* = 0.77). Wang et al. (2022a) collected wheat canopy spectral data via UAV and applied nine machine learning algorithms to estimate chlorophyll content. The developed models achieved r=0.63, RMSE=3.28, and NRMSE=16.2% under normal irrigation, and r=0.63, RMSE=3.47, and NRMSE=19.2% under drought stress. Liu et al. (2021) collected multispectral images and, using determination coefficients, developed multiple stepwise regression, partial least squares regression, and artificial neural network (ANN) models to estimate wheat canopy LAI and chlorophyll content (SPAD) from UAV data at different flight altitudes. The ANN model achieved the highest accuracy for chlorophyll estimation, with an *R²* of 0.804 and RMSE of 0.135. Wang et al. (2022b) used multispectral remote sensing images to obtain leaf area index (LAI) and flag leaf chlorophyll content (CC) under normal irrigation and drought stress. They applied classification and regression trees (CART) combined with cross-validation to estimate LAI and CC comprehensively. QTL mapping was performed based on the analysis of predicted and measured values. Results showed that the coefficient of determination (*R²*) ranged from 0.79 to 0.93, root mean square error (RMSE) ranged from 0.39 to 1.05, relative error (RE) ranged from 0.19 to 0.18, and RMSE ranged from 0.16 to 1.16. Cheng et al. (2017) developed a univariate regression model and a support vector machine regression model for apple tree leaves using nine color parameters; the support vector machine regression model was more accurate than the univariate regression model (*R^2^* = 0.83 and RMSE = 0.03).

The current use of UAVs during crop production is mainly focused on monitoring and inverting the crop growth status. However, the high-throughput mining for trait-related genes based on phenotypes derived from an inversion model and high-throughput SNP array data has rarely been reported for wheat. In the present study, we constructed a wheat chlorophyll content model by predicting the artificial chlorophyll contents of 119 wheat samples and combining the data with multispectral UAV parameters to predict wheat chlorophyll contents. A genome-wide association analysis of the predicted wheat chlorophyll contents and the predicted artificial chlorophyll contents was performed and the feasibility of using spectral information to predict wheat chlorophyll contents was assessed.

## 2 Materials and methods

### 2.1 Plant material

A total of 119 wheat materials were analyzed. The experimental group was divided into three subgroups(Contains 16 landraces (lines),35 domestic and foreign imported varieties (lines) and 68 self-bred varieties (lines)) (Schedule 1). Relevant representative information was provided by the Xinjiang Agri-cultural University Wheat Research Group. The experimental samples included excellent wheat resources from Xinjiang as well as domestic wheat and imported wheat germplasm. The Great Frontier Elf 4 drone (Multispectral Edition) selected for this study has red(650nm ± 16nm), red edge(730nm ±16nm), near-infrared(840nm ± 26nm), blue(450nm ± 16nm), green(560nm ±16nm), and visible light multispectral lenses.

### 2.2 Test methods

#### 2.2.1 Experimental design

In fall 2019, test materials were grown in Zepu County (77.3° E, 38.24° N; E1), south of Tianshan, Xinjiang, and in Manas County (86.25° E, 44.30° N; E2), north of Tianshan, Xin-jiang. The following two treatment methods were implemented: standard treatment (normal watering throughout the growth period); drought stress treatment, during which a randomized group design was used to expose experimental materials to drought stress (watering was restricted during the Heading, Flowering, and Grain Filling Stages). Both treatments were completed using two replicates. Each cultivar was grown three rows apart (0.2 m). Each row measured 3 meters (m) in length and had a seeding capacity of 240 grains per row. The total cultivated area was 7 mu, which is equivalent to approximately 0.4667 hectares (ha).

#### 2.2.2 Collection of multispectral data

The Great Frontier Genie 4 drone (Multispectral Edition) was used for multi-spectral image data acquisition (12:00–16:00) on a clear and cloudless day during the wheat Heading Stage, Flowering Stage, and Grain Filling Stage. The first drone mission took place on a sunny day. The drone was fixed at an altitude of 12 m, with a heading overlap of 75% and a lateral overlap of 70%. Spatial resolution of approximately 0.926 (cm/pixel).The Photo Interval mode was used at 2 s intervals, with the main dish set parallel to the solar incidence angle.

#### 2.2.3 Determination of chlorophyll contents

Field measurements were conducted using a portable SPAD-502 Plus chlorophyll meter to analyze wheat flag leaf chlorophyll contents. For each of the cultivars (lines), five normally growing plants were examined. The mean leaf chlorophyll content of the five plants per cultivar (line) was calculated as one replicate. All phenotype data were collected within 3 days of the UAV mission. The mean chlorophyll content for two replicates was recorded (**Table 1**).

**Table 1:** Manas UAV multispectral imagery and SPAD data acquisition program.

Collection time	Fertility period	Collection data
2021.5.8	Heading Stage	SPAD+UAV
2021.5.21	Flowering	SPAD+UAV
2021.5.28	Grain Filling	SPAD+UAV

#### 2.2.4 Phenotypic analysis

Drone images were combined using the PIX 4D software package, with the five major bands (red, red edge, near-infrared, blue, and green light) combined one at a time using the Agricultural Multispectral model (Li and Shi, 2020). Images were generated using the 4D exponential PIX calculator in the single band emissivity range. The regional pixel reflectance of the five main bands in wheat images was extracted using ENVI 4.1 and spectral information was obtained for all five main bands. Excel 2007 and QTL IciMapping v4.1 were used to process and calculate the data for the entire experimental period. The chlorophyll content was manipulated using extreme values, mean values and standard deviations, variance factors, and generalized genetic strengths: h_B_^2^ = σ_g_^2^/(σ_g_^2^ + σ_e_^2^) (Mosleth et al., 2020), with σ_g_^2^ and σ_e_^2^ representing genetic variance and environmental variance, respectively.

#### 2.2.5 Selection of vegetation indices

Vegetation indices, derived from the combined reflectance of different spectral bands, can mitigate the influence of background soil factors on plant spectra and increase the accuracy of estimated chlorophyll contents. In this study, Within the Python environment utilizing NumPy, Pandas, Matplotlib, and Seaborn packages, the importance of 18 vegetation in-dices was evaluated using the random forest algorithm, after which the most relevant in-dices were selected on the basis of their correlation with SPAD values. The selected vegetation indices were then used to model and predict SPAD values. Formulas for calculating vegetation indices are provided in **Table 2**.

**Table 2:** Vegetation index and its calculation formula.

Vegetation Index	Formula To Calculate	Reference
NDVI	NDVI=(RNir−RRed)/(RNir+RRed)	(Schnell, 1974)
GNDVI	$GNDVI=(R_{Nir}-R_{Green})/(R_{Nir}+R_{Green})$	(Wagner, 1996)
NGBDI	$NGBDI=(R_{Green}-R_{Blue})/(R_{Green}+R_{Blue})$	(Hunt et al., 2005)
NGRDI	NGRDI=(RGreen−RRed)/(RGreen+RRed)	(Hunt et al., 2005)
RERDVI	$RERDVI=(R_{Nir}-R_{Red\_edge})/(R_{Nir}+R_{Red\_edge})$	(Kim et al., 1994)
SAVI	$SAVI=2.5*(R_{Nir}-R_{Red})/(R_{Nir}+R_{Red}+0.5)$	(Huete, 1988)
GOSAVI	$GOSAVI=1.16*[(R_{Nir}-R_{Green})/(R_{Nir}+R_{Green}+0.16)]$	(Gilabert et al., 2002)
REOSAVI	$REOSAVI=1.16*[(R_{Nir}-R_{Red})/(R_{Nir}+R_{Red}+0.16)]$	(Kim et al., 1994)
OSAVI	$OSAVI=(R_{Nir}-R_{Red})/(R_{Nir}+R_{Red}+0.16)$	(Rondeaux et al., 1996)
RVI	$RVI=R_{Nir}/R_{Red}$	(Pearson and Miller, 1972)
DVI	$DVI=R_{Nir}-R_{Red}$	(Tucker, 1979)
GRVI	$GRVI=R_{Nir}/R_{Green}$	(Tucker, 1979)
EXG	$EXG=2R_{Green}-R_{Red}-R_{Blue}$	(Torres-Sánchez et al., 2014)
TVI	$TVI=0.5[120(R_{Nir}-R_{Green})-200(R_{Red}-R_{Green})]$	(Broge and Leblanc, 2001)
CARI	$CARI=(R_{Red\_edge}-R_{Red})/0.2*(R_{Red\_edge}+R_{Red})$	(Rondeaux et al., 1996)
VARIgreen	$VARIgreen=(R_{Green}-R_{Red})/(R_{Green}+R_{Red}-R_{Blue})$	(Gitelson et al., 2002)
VARIred	$\begin{array}{l} VARIred=(R_{Red\_edge}-1.7*R_{Red}+0.7*R_{Blue})/\\ \left(R_{Red\_edge}+2.3*R_{Red}-1.3*R_{Blue}\right) \end{array}$	(Gitelson et al., 2002)
EVI	$\begin{array}{l} EVI=2.5*(R_{Nir}-R_{Red})/\\ \left(R_{Nir}+6*R_{Red}-7.5*R_{Blue}+1\right) \end{array}$	(Bolton and Friedl, 2013)

$R_{Blue}$ .、 $R_{Green}$ 、 $R_{Red}$ 、 $R_{Red\_edge}$ 、 $R_{Nir}$ respectively represent the reflectance of blue wave band, green band, red band, red edge band and near infrared band.

#### 2.2.6 Development of a chlorophyll content model

The BP neural network is a supervised learning algorithm. Its core concept involves analyzing the error between the results obtained from training and the expected outcomes. Weights and thresholds are subsequently adjusted to gradually decrease the error, ultimately resulting in a model where the output is closely aligned with the desired results.

A deeper architecture typically includes: 1 input layer (number of input features), 1 hidden layers (to capture complex relationships), and 1 output layer (for predicting chlorophyll content), 18 neurons per layer, Learning rate 0.001, number of 50 epochs. The ratio for dividing the training and validation data is 70%:30%.

Chlorophyll contents determined manually were used in two environments under two treatment conditions (normal irrigation and drought) for a total of eight replicates. For the manually calculated chlorophyll contents, eight iterations were computed at the 70% level of the test set. The validation set was modeled and validated using data for 84 randomly selected samples (Zhou et al., 2020) as a training dataset and data for 35 samples as a validation dataset. The *R^2^*, RMSE, and relative error (RE) values were used to determine the correlation between the predicted values of the model. Generally, if *R^2^* is close to 1, the RMSE and RE values are relatively low, reflecting the accuracy of model predictions. Using Python’s NumPy, Pandas, and scikit-learn packages to construct a BP neural network. Extracted spectral information was distributed using a neural network. A deep learning model predicted chlorophyll content using UAV-derived spectral indices This model was implemented by the Xinjiang Agricultural University Engineering Research Center for High-quality Special Wheat Crops.

#### 2.2.7 Genome-wide association analysis

We used the Tassel v5.0 software package to analyze the association between the predicted and actual chlorophyll contents under normal irrigation and drought treatment conditions and SNP markers using the mixed linear model (MLM) Q + K (Yang et al., 2017). After the chipset was introduced, the Q value (calculated using Software 2.3), phenotype data (chlorophyll content), and genetic relationship were analyzed. Calculation results were obtained and exported to screen for genes. The threshold for determining significant correlations between markers and traits was set at P< 0.001 (Arif and Brner, 2021). In terms of the LD decay distance, markers that were significantly close to each other (less than the LD decay distance) were ultimately merged at a particular locus. The physical location of a marker was entered online (https://urgi.versailles.inrae.fr/blast_iwgsc/blast.php) (Gebrewahid et al., 2020) for the subsequent search and comparison of chlorophyll contents.

## 3 Results

### 3.1 Spectroscopic drone data

A combined image was produced using PIX 4D to capture 36 multispectral reflections of two environments and three time periods, each with an RGB (visible light) image and red, red edge, near-infrared, blue, and green reflectance images. Pixel reflectance in 30 single-band images was extracted using ENVI 4.1 software, and 16,960 DN values were obtained. Under normal irrigation conditions, the average red, red edge, near-infrared, green, and blue light reflectance rates were 7.68%, 34.03%, 56.84%, 15.93%, and 6.74%, respectively (Heading Stage); 7.65%, 33.11%, 55.14%, 15.06%, and 6.38%, respectively (Flowering Stage); and 5.85%, 27.22%, 37.47%, 7.70%, and 4.54%, respectively (Grain Filling Stage). Under drought conditions, the average red, red edge, near-infrared, green, and blue light reflectance rates were 7.88%, 33.74%, 55.40%, 15.82%, and 6.70%, respectively (Heading Stage); 7.70%, 31.42%, 52.33%, 14.81%, and 6.38%, respectively (Flowering Stage); and 6.95%, 27.88%, 34.93%, 8.31%, and 4.94%, respectively (Grain Filling Stage) (**Table 3**). The whiteboard DN value was 65,000 with 99.8% reflectivity. The reflectivity of the five bands varied between 4.54% and 56.84%. Drought stress results in decreases in biomass and leaf area index, which in turn increase the overall reflectivity of band images associated with vegetation in arid regions.

**Table 3:** Reflectance of spectral bands in wheat under normal irrigation (W) and drought (D) across growth stages.

Treatment	Band	HS (%)	FL (%)	GF (%)
Water	Red	7.68	7.65	5.85
	R-E	34.03	33.11	27.22
	Nir	56.84	55.14	37.47
	Green	15.93	15.06	7.7
	Blue	6.74	6.38	4.54
Drought	Red	7.88	7.7	6.95
	R-E	33.74	31.42	27.88
	Nir	55.4	52.33	34.93
	Green	15.82	14.81	8.31
	Blue	6.7	6.38	4.94

HS, Heading Stage; FL, Flowering; GF, Grain filling; Red, Red light; Red-Edge, The red edge light; Nir, near-infrared; Green, Green light; Blue, Blue light.

**Figure 1:**
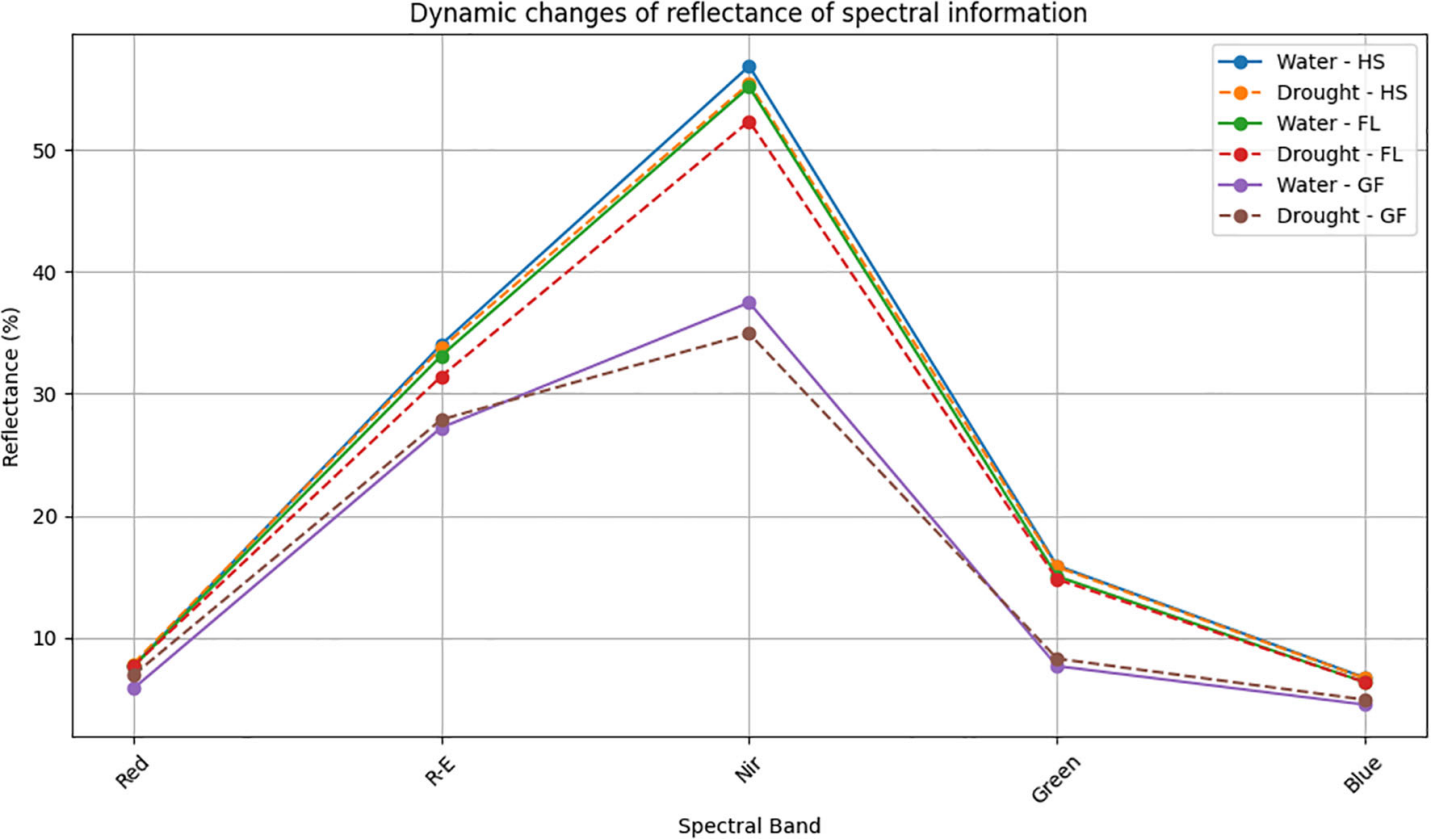
Dynamic changes of reflectance of spectral information. HS, Heading Stage; FL, Flowering; GF, Grain filling; Red, Red light. Water: normal irrigation; Drought: drought stress. R-E: The red edge light; Nir: near-infrared; Green: Green light; Blue: Blue light.

For the five principal bands, the rank order for the light reflectance of wheat exposed to two treatment conditions was as follows: near-infrared light > red edge light > green light > red light > blue light. Under two different treatment conditions, there was a de-creasing trend in light reflectance during the Heading, Flowering, and Grain Filling Stages. The rank order of reflectance among stages was as follows: Heading > Flowering > Grain Filling (**Figure 1**). Under normal irrigation conditions, red, red edge, near-infrared, green, and blue light reflectance decreased by 0.37%, 2.72%, 2.99%, 5.51%, and 5.3%, respectively, during the Flowering Stage (relative to the levels in the Heading Stage). red, red edge, near-infrared, green, and blue light reflectance decreased by 23.54%, 17.76%, 32.05%, 48.87%, and 28.88%, respectively, during the Grain Filling Stage (relative to the levels in the Flowering Stage). Under drought conditions, red, red edge, near-infrared, green, and blue light reflectance decreased by 2.31%, 6.89%, 5.54%, 6.38%, and 4.80%, respectively, during the Flowering Stage (relative to the levels in the Heading Stage). red, red edge, near-infrared, green, and blue light reflectance decreased by 9.72%, 11.27%, 33.25%, 43.87%, and 22.75%, respectively, during the Grain Filling Stage (relative to the levels in the Flowering Stage). The decrease in reflectance across the five spectral bands ranged from 17.78% to 48.87%. In wheat, the reflectance in the red band during the heading, flowering, and grain filling stages was lower under normal irrigation conditions than under drought conditions. The reflectance in the blue band during the heading and Flowering Stages did not differ significantly between the two treatment conditions; however, the reflectance in the blue band during the Grain Filling Stage was lower under normal irrigation conditions than under drought conditions. By contrast, the reflectance in the near-infrared band was higher un-der normal irrigation conditions than under drought conditions across all growth stages. Reflectance was lowest for the blue and red bands, implying that wheat plants primarily absorb blue and red light during the heading, flowering, and grain filling stages.

### 3.2 Optimal vegetation index

Vegetation indices are often selected on the basis of empirical values, with limited visualization of the selection process. In this study, a random forest algorithm was used to as-sess the contribution of vegetation indices to SPAD values during the heading, flowering, and grain filling stages of wheat plants that underwent normal irrigation and drought treatments. Additionally, correlations between spectral parameters in three growth stages and winter wheat SPAD values were analyzed. The optimal vegetation index, which was identified by integrating the results of both analyses, was then included in a BP neural network for model inversion and prediction.

**Figure 2:**
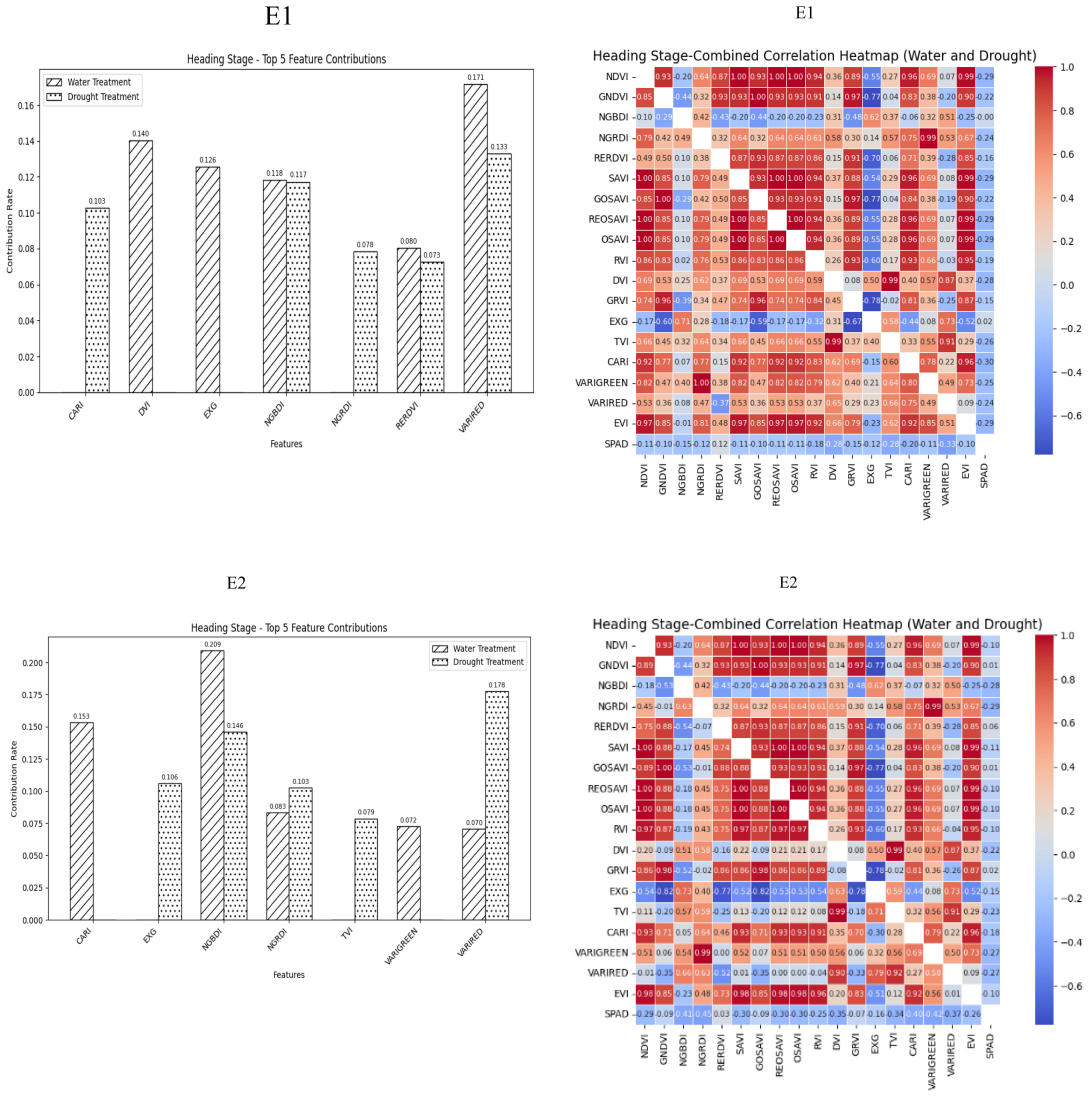
Distribution of contribution rate of vegetation index to SPAD under flood and drought treatment at Heading Stage of winter wheat and correlation between different vegetation indexes and SPAD of winter wheat under flood and drought treatment at Heading Stage of winter wheat On the correlation heat map, it is normal irrigation treatment, and the next is drought stress treatment. E1: Southern Xinjiang; E2: Northern Xinjiang. Note: All vegetation indices that appear in the figure are explained in **Table 2**.

During the Heading Stage, most spectral vegetation indices selected under normal irrigation and drought conditions reached a highly significant level. The contributions of vegetation indices to SPAD values during this stage under different treatment conditions indicated NGBDI, RERDVI, and VARIRED were among the top five indices. Additionally, the correlation heatmap revealed that the correlations were strongest for CARI under normal irrigation conditions (*R^2^* = -0.3) and VARIRED under drought conditions (*R^2^* = −0.33). Hence, the vegetation indices contributing to the E1 environmental model inversion were NGBDI, RERDVI, VARIRED and CARI. Similarly, the vegetation indices for the E2 environmental model inversion included NGBDI, NGRDI, and VARIRED (**Figure 2**).

**Figure 3:**
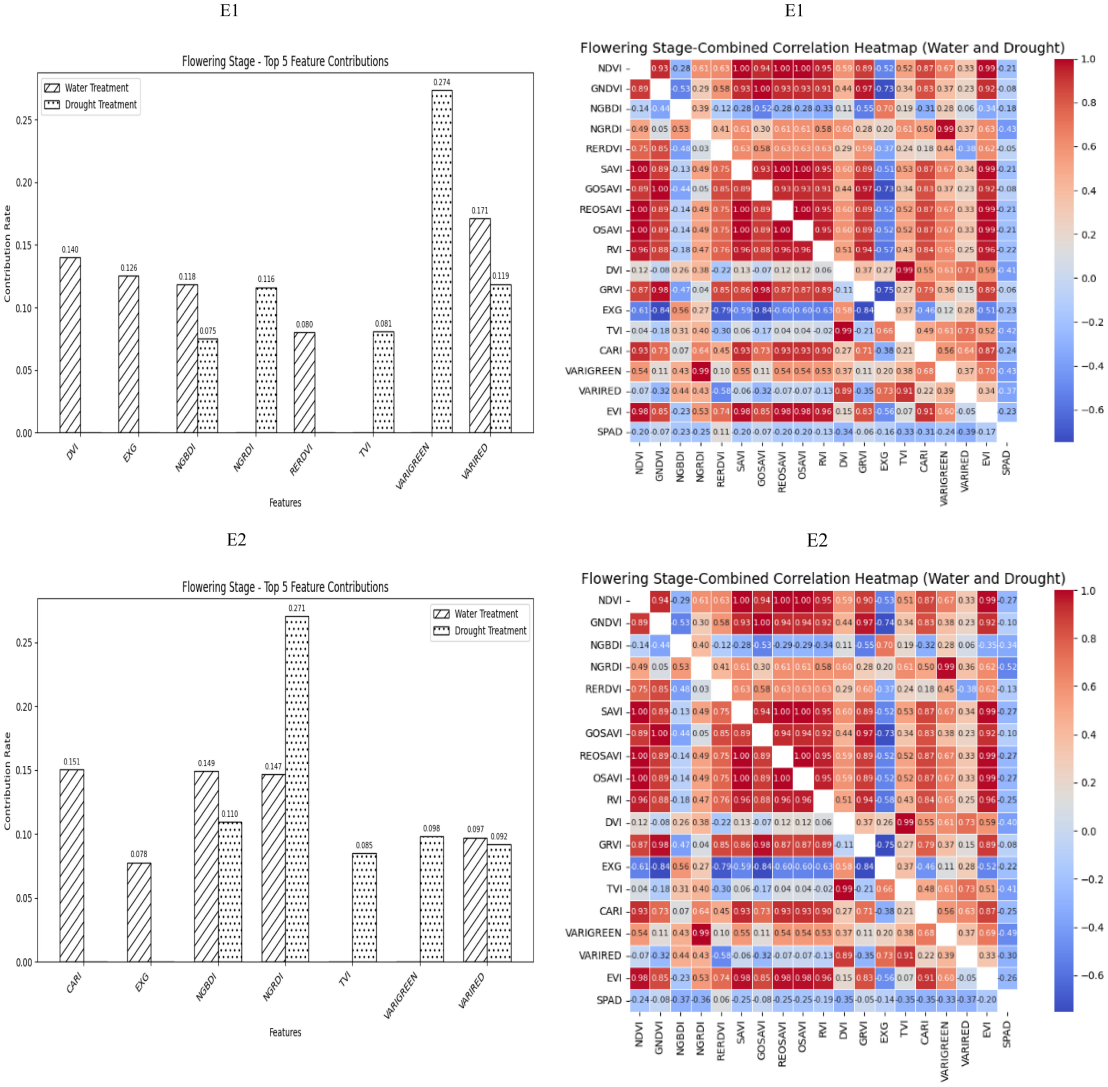
The distribution of the contribution rate of vegetation index to SPAD under flood and drought treatment at the Flowering Stage of winter wheat and the correlation between different vegetation indexes and SPAD of winter wheat under flood and drought treatment at the Flowering Stage of winter wheat. On the correlation heat map, it is normal irrigation treatment, and the next is drought stress treatment. E1: Southern Xinjiang; E2: Northern Xinjiang. Note: All vegetation indices that appear in the figure are explained in **Table 2**.

During the Flowering Stage, most of the selected spectral vegetation indices under both normal irrigation and drought conditions reached a highly significant level. According to their contributions to SPAD values, NGBDI and VARIRED were among the top five vegetation indices under different treatment conditions. The correlation heatmap showed that the correlations were strongest for NGRDI and VARIGREEN under normal irrigation conditions (*R^2^* = −0.43) and VARIRED under drought conditions (*R^2^* = −0.39). Therefore, the vegetation indices contributing to the E1 environmental model inversion were NGBDI, VARIRED, NGRDI and VARIGREEN. Similarly, the vegetation indices for the E2 environmental model inversion included NGBDI, NGRDI, and VARIRED (**Figure 3**).

**Figure 4:**
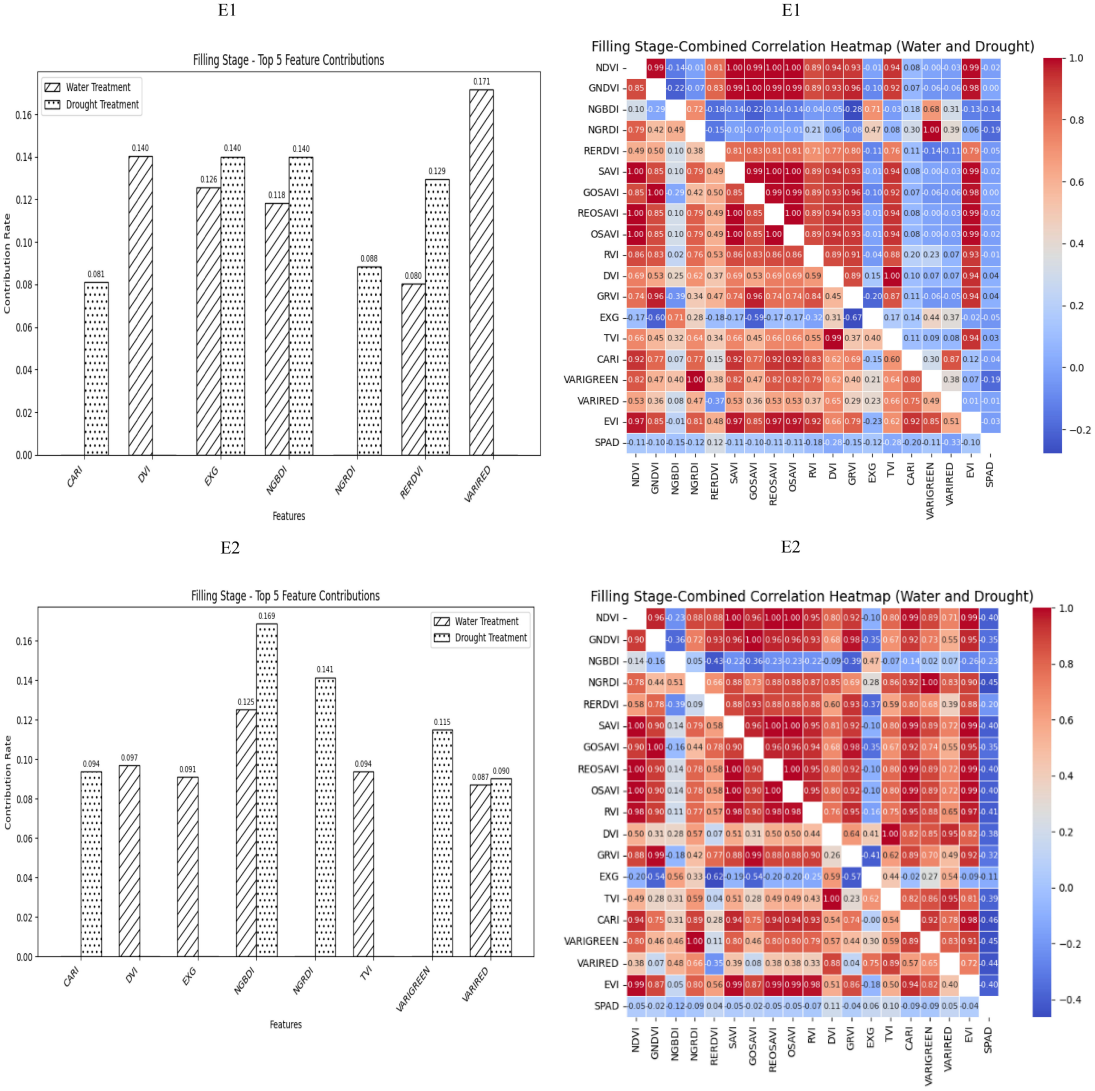
The distribution map of the contribution rate of vegetation index relative to SPAD under paddy and drought treatment during the filling stage of winter wheat and the correlation map of different vegetation indexes and winter wheat SPAD under paddy and drought treatment during the Flowering Stage of winter wheat. On the correlation heat map, it is normal irrigation treatment, and the next is drought stress treatment. E1: Southern Xinjiang; E2: Northern Xinjiang. Note: All vegetation indices that appear in the figure are explained in **Table 2**.

During the grain filling stage, most of the selected spectral vegetation indices under both normal irrigation and drought conditions reached a highly significant level. On the basis of the contributions of these vegetation indices to SPAD values, EXG, NGBDI, VARIRED was among the top five indices under different treatment conditions. The correlation heatmap indicated that the correlations were strongest for VARIGREEN and NGRDI under normal irrigation conditions (*R^2^* = −0.19) and VARIRED under drought conditions (*R^2^* = −0.33). Consequently, the vegetation indices contributing to the E1 environmental model inversion were VARIRED, EXG, NGBDI, CARI, VARIGREEN and NGRDI. Similarly, the vegetation indices for the E2 environmental model inversion included NGBDI, VARIRED, and CARI (**Figure 4**).

### 3.3 Artificially measured values and predicted phenotypes

**Figure 5:**
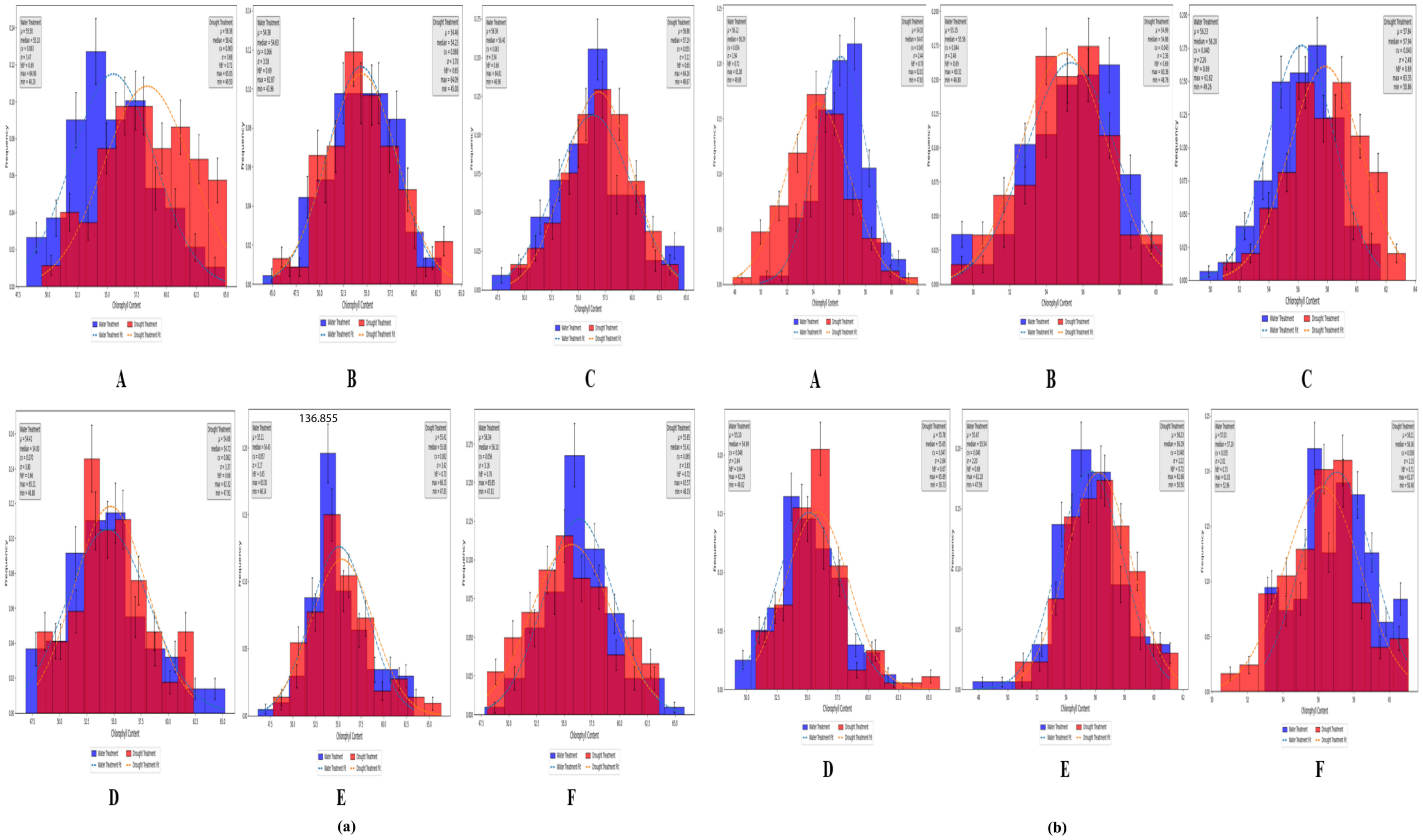
SPAD distribution map of Zepu and Manas winter wheat at different growth stages **(a)** The measured SPAD values of Zepu and Manas winter wheat; **(b)** SPAD prediction value of Zepu and Manas winter wheat. **(A–C)** represent the statistical analysis chart of SPAD content at Heading Stage, Flowering Stage and filling stage of Zepu environment under normal irrigation and limited water treatment. **(D–F)** represent the statistical analysis map of SPAD content at Heading Stage, Flowering Stage and filling stage in Manas environment under normal irrigation and limited water treatment.

According to the measured values under both treatment conditions, the chlorophyll content of the analyzed wheat plants varied during the heading, flowering, and grain filling stages (**Figure 5a**), although the variations were not substantial. The measured SPAD values were analyzed in terms of the following: mean (μ), median, coefficient of variation (CV), standard deviation (σ), heritability (h_B_^2^), maximum (max), and minimum (min). As shown in sections A, B, and C of **Figure 5a**, at the Zepu study site, under normal irrigation conditions, μ was 54.38–56.36, with median values between 54.63 and 56.40, CV ranging from 6.2% to 6.6%, σ between 3.42 and 3.58, h_B_^2^ from 0.66 to 0.69, max between 62.87 and 64.90, and min from 43.96 to 48.20. Under drought conditions, μ was 54.46–58.24, with median values between 54.22 and 58.30, CV ranging from 5.5% to 6.8%, σ between 3.12 and 3.7, h_B_^2^ from 0.65 to 0.71, max between 64.09 and 65.05, and min from 45.00 to 49.50. Similarly, sections D, E, and F of **Figure 5a** indicate that at the Manas study site, μ, median, CV, σ, h_B_^2^, max, and min varied across three growth stages and under two treatment conditions, but these differences do not need be elaborated further.

A model was developed using neural networks to predict chlorophyll contents through distributed computation. Under both normal irrigation and drought conditions, the predicted chlorophyll content of wheat differed among the heading, flowering, and grain filling stages (**Figure 5b**). The predicted SPAD values were analyzed in terms of μ, median, CV, σ, h_B_^2^, max, and min. As demonstrated in sections A, B, and C of **Figure 5b**, at the Manas study site, under normal irrigation conditions, μ was 55.35–56.22, with median values between 55.56 and 56.29, CV ranging from 3.4% to 4.4%, σ from 1.94 to 2.46, h_B_^2^ from 0.69 to 0.72, max between 60.31 and 61.92, and min from 48.80 to 49.89. Under drought conditions, μ was 54.35–57.84, with median values between 54.47 and 57.94, CV from 4.3% to 4.5%, σ between 2.36 and 2.48, h_B_^2^ from 0.69 to 0.70, max between 60.36 and 63.35, and min from 47.83 to 50.86. Similarly, sections D, E, and F of **Figure 5b** indicate that at the Manas study site, μ, median, CV, σ, h_B_^2^, max, and min varied across three growth stages and under two treatment conditions, but this diversity does not need be elaborated further.

Overall, the data were widely distributed and highly variable, reflecting significant variations in SPAD values during the heading, flowering, and grain filling stages as well as the rich genetic diversity within the population. The predicted values were more conservative than the measured values. More specifically, the maximum and minimum predicted values were lower and higher, respectively, than the measured values.

For both the normal irrigation and drought treatments at the two study sites, the variation range of the measured values showed that during the Heading Stage, the drought treatment varied from the normal irrigation treatment by 0.1% and −0.8%, while in the Flowering Stage, the drought treatment varied from the normal irrigation treatment by 0.2% and 0.5%. In the grain filling stage, variations of −0.8% and 1% were revealed for the drought treatment (compared with the normal irrigation treatment).

In terms of the variation range of the predicted values, during the Heading Stage, the drought treatment varied from the normal irrigation treatment by 1.1% and −0.1%. In the Flowering Stage, variations of −0.1% and 0% were revealed for the drought treatment (compared with the normal irrigation treatment). In the grain filling stage, the drought treatment varied from the normal irrigation treatment by 0.3% in both instances. The variation range increased from the Heading Stage to the Flowering Stage, but gradually decreased as the growth period was extended.

### 3.4 Analysis of the correlation between measured and predicted values

An analysis of the chlorophyll contents predicted via manual measurement and the in-version model showed that the correlation between the predicted and measured values was between 0.90 and 0.93 at the Heading Stage, with an *R^2^* value of 0.80–0.87, under normal irrigation conditions. The correlation between the predicted and measured values in the Flowering Stage ranged from 0.91 to 0.92 (*R^2^* = 0.83–0.84). During the grain filling stage, the correlation between the predicted and measured values was 0.88–0.90 (*R^2^* = 0.77–0.81). For the drought-treated samples, the correlation between the predicted and measured values was 0.57–0.70 (*R^2^* = 0.32–0.49). In the Flowering Stage, the correlation between the predicted and measured values ranged from 0.89 to 0.91 (*R^2^* = 0.79–0.83). The correlation between the predicted and measured values in the Grain Filling Stage was be-tween 0.94 and 0.96 (*R^2^* = 0.88–0.91). The correlations in the overall data reached 0.87, with a coefficient of determination of 0.75. Thus, the artificial neural network-based chlorophyll content model can reliably predict chlorophyll contents in the two environments analyzed in this study. The correlation between the predicted and measured values of the model in the Heading Stage under drought conditions was lower than that in the other stages and under normal irrigation conditions (**Table 4**).

**Table 4:** Correlation analysis between predicted values and measured values.

Environment	Phase	Control	R	R^2^	RMSE	RE
E1	HS	W	0.90^**^	0.80	0.18	0.03
		D	0.70^**^	0.49	0.24	0.04
	FL	W	0.92^**^	0.84	0.14	0.02
		D	0.89^**^	0.79	0.18	0.03
	GF	W	0.90^**^	0.81	0.15	0.02
		D	0.94^**^	0.88	0.16	0.03
E2	HS	W	0.93^**^	0.87	0.16	0.03
		D	0.57^**^	0.32	0.28	0.04
	FL	W	0.91^**^	0.83	0.14	0.02
		D	0.91^**^	0.83	0.17	0.03
	GF	W	0.88^**^	0.77	0.16	0.02
		D	0.96^**^	0.91	0.24	0.03

W, Water; D, Drought; HS, Heading Stage; FL, Flowering; GF, Grain filling; E1, Southern xinjiang; E2, Northern xinjiang.

The symbol ** indicates "significant correlation at the 1% significance level," implying highly reliable results.

### 3.5 Marker–trait correlation analysis

A total of 36,873 SNP markers selected from a 50K chip were combined with the predicted chlorophyll contents of 119 experimental materials for a genome-wide association analysis. We controlled false positives due to the population structure and kinship and used the MLM Q + K mixed model. Applying a threshold of<0.001, we identified 308 loci across 21 chromosomes that explained 7.58%–19.58% of the phenotypic variation. Specifically, the association analysis performed on the basis of the predicted chlorophyll contents revealed 206 loci that explained 7.58%–19.58% of the phenotypic variation. By contrast, the analysis conducted on the basis of measured values identified 102 loci that explained 9.31%–15.83% of the phenotypic variation. The detailed distribution of the 308 loci under different treatment conditions and at different time points is presented in **Table 5**.

**Table 5:** Correlation analysis site comparison between predicted values and measured values.

Treatment	Source	HS			FL			GF		
		Site number	P-value	R^2^ (%)	Site number	P-value	R^2^ (%)	Site number	P-value	R^2^ (%)
W	measured	12	9.91E-05	9.42~14.56	46	1.96E-04	9.61~13.01	14	1.87E-04	10.04~13.56
	predicted	12	1.26E-04	9.65~13.42	52	7.63E-06	9.22~18.98	45	1.05E-05	9.44~18.24
D	measured	10	2.87E-04	9.31~11.98	8	1.51E-04	10.12~15.83	12	2.96E-04	9.83~13.02
	predicted	32	1.08E-04	9.14~12.68	42	6.77E-05	7.58~14.03	23	5.77E-06	10.04~19.58

W, Water; D, Drought; HS, Heading Stage; FL, Flowering; GF, Grain filling.

An association analysis of the real and predicted data detected 18 loci distributed on chromosomes 1A, 1B, 2B, 3B, 4B, 5A, 5B, 5D, 6B, 6D, 7A, and 7B, which explained 7.58%–19.58% of the phenotypic variation(**Table 6**). The P-value was 9.91 × 10^−5^–9.90 × 10^−4^ for chloro-phyll-related loci (mean of 6.71 × 10^−4^), which accounted for 9.31%–15.83% of the phenotypic variation (mean of 10.82%). The P-value was 5.77 × 10^−6^–1.00 × 10^−3^ (mean of 4.25 × 10^−4^) for the chlorophyll content of the solution. The proportion of the phenotypic variation explained ranged from 7.58% to 19.58% (mean of 11.50%).

**Table 6:** Correlation analysis of measured and predicted values and statistics of overlap sites.

Source	Marker	Chr	Pos	p	MarkerR2	Candidate genes	Gene annotation
Measured	*AX-94849392*	1A	6155249	4.78E-04	10.91%	TraesCS1A01G009900	Disease resistance protein (NBS-LRR class) family
Predict	*AX-94492529*	1A	3118443	7.13E-04	9.65%	TraesCS1A01G005400	APOLLO
Measured	*AX-94620350*	1B	50183860	8.79E-04	10.13%	TraesCS1B01G065900	Arginine/serine-rich splicing factor, putative
Predict	*AX-95175396*	1B	48568925	6.77E-04	9.88%	TraesCS1B01G064000	serine hydroxymethyltransferase 2
Measured	*AX-179557826*	2B	765138788	5.79E-04	11.01%	null	null
Predict	*AX-179557826*	2B	765138788	8.28E-04	10.03%	null	null
Measured	*AX-89366787*	3B	8859867	8.02E-04	9.72%	null	null
Predict	*AX-111019347*	3B	11947067	7.69E-04	10.20%	TraesCS3B01G027600	SKP1-like protein
Measured	*AX-95661558*	3B	737861984	8.02E-04	9.72%	null	null
Predict	*AX-94494956*	3B	730345453	5.02E-04	8.52%	TraesCS3B01G484000	Beta-glucosidase, putative
Measured	*AX-179560086*	3B	759703505	5.18E-04	10.46%	null	null
Predict	*AX-112287513*	3B	757299512	1.08E-04	12.68%	null	null
Measured	*AX-111595357*	4B	665552447	6.34E-04	10.26%	TraesCS4B01G388900	DNA (Cytosine-5-)-methyltransferase
Predict	*AX-94552601*	4B	666571859	1.29E-04	12.61%	TraesCS4B01G602100LC	DNA (Cytosine-5-)-methyltransferase
Measured	*AX-95659156*	5A	422741070	3.55E-04	11.41%	TraesCS5A01G323800LC	Protein FAR1-RELATED SEQUENCE 5
Predict	*AX-95659156*	5A	422741070	1.73E-04	11.81%	TraesCS5A01G323800LC	Protein FAR1-RELATED SEQUENCE 5
Measured	*AX-110598576*	5A	576774974	3.52E-04	11.97%	TraesCS5A01G379400	Chalcone synthase
Predict	*AX-110618351*	5A	577711097	3.00E-04	12.09%	TraesCS5A01G527900LC	Eukaryotic translation initiation factor 3 subunit C-like protein
Measured	*AX-109475699*	5B	644853755	9.45E-04	11.24%	TraesCS5B01G471300	Beta purothionin
Predict	*AX-111522577*	5B	643465238	2.77E-04	10.95%	TraesCS5B01G679600LC	Nipped-B-like protein A
Measured	*AX-110916065*	5D	560023418	7.72E-04	9.48%	TraesCS5D01G557800	Receptor-like protein kinase
Predict	*AX-89322127*	5D	560460920	4.18E-04	10.92%	TraesCS5D01G655500LC	Flavin-containing monooxygenase
Measured	*AX-110126169*	6B	121717558	8.55E-04	10.48%	TraesCS6B01G185900LC	Endonuclease/exonuclease/phosphatase family protein
Predict	*AX-109815710*	6B	122944348	7.63E-06	18.98%	TraesCS6B01G187200LC	Endonuclease/exonuclease/phosphatase family protein
Measured	*AX-109866357*	6D	467023050	9.90E-04	9.83%	TraesCS6D01G508400LC	Serine/threonine-protein kinase
Predict	*AX-110434749*	6D	470942157	4.16E-05	14.94%	TraesCS6D01G402600	Receptor-like kinase
Measured	*AX-111530810*	7A	198197404	9.79E-04	9.70%	TraesCS7A01G227800	Ribonucleoside-diphosphate reductase
Predict	*AX-109283182*	7A	198787726	7.20E-04	8.16%	TraesCS7A01G228300	Carboxyl-terminal-processing protease
Measured	*AX-95630787*	7A	669185532	8.49E-04	10.48%	null	null
Predict	*AX-94567102*	7A	666606185	9.67E-04	9.68%	TraesCS7D01G457700	Aspartyl/glutamyl-tRNA(Asn/Gln) amidotransferase subunit B, putative isoform 2
Measured	*AX-111627821*	7B	333420288	6.49E-04	12.33%	TraesCS7B01G194000	Epoxide hydrolase 2
Predict	*AX-111569286*	7B	331619956	6.65E-05	15.21%	TraesCS7B01G341100LC	28S ribosomal S34
Measured	*AX-111472616*	7B	337472880	4.92E-04	10.70%	TraesCS7B01G196400	Serine/threonine-protein kinase
Predict	*AX-111668293*	7B	337173355	4.61E-04	9.35%	TraesCS7B01G344800LC	Protein FAR1-RELATED SEQUENCE 5
							
Measured	*AX-111629552*	7B	601004348	1.51E-04	14.96%	TraesCS7B01G576100LC	calcium-dependent lipid-binding family protein
Predict	*AX-110928740*	7B	600838345	5.00E-04	11.35%	TraesCS7B01G344900	Transmembrane protein, putative

**Figure 6:**
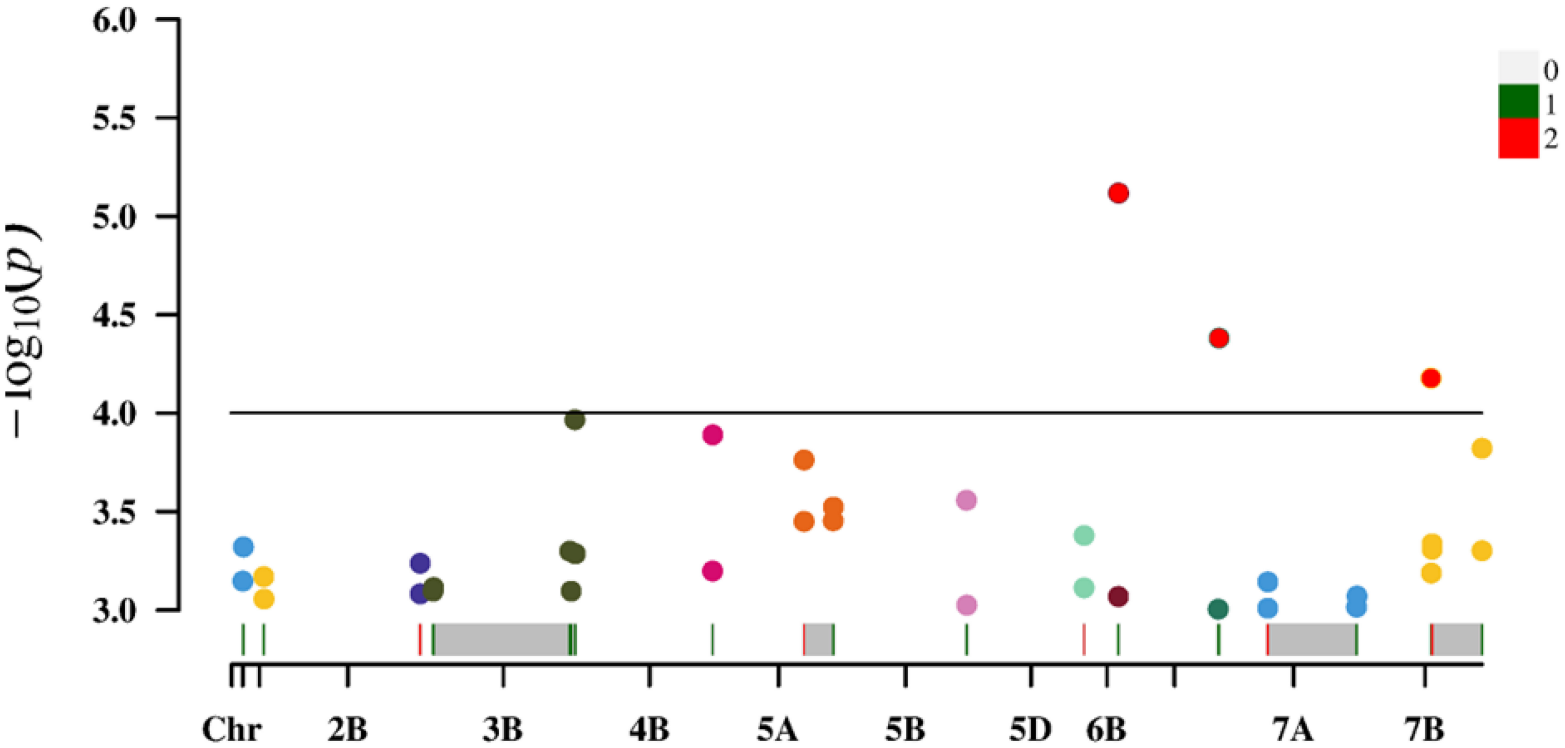
Manhattan plot of 18 overlapping loci/sites.

For the loci identified using both measured and predicted values, the average P-value for predicted chlorophyll contents was relatively small, indicating a strong association. Additionally, the average phenotypic variation explained (%) was relatively high, reflecting the high quality of the loci detected for the predicted chlorophyll contents (**Figure 6**).

### 3.6 Candidate loci for predicting wheat flag leaf chlorophyll contents

We analyzed microarray data for 90K wheat SNPs by combining 308 loci based on predicted and measured values, with a genome-wide association analysis conducted to identify loci significantly associated with the chlorophyll content. The common wheat Chinese Spring Genome Database was searched for the detected loci. Candidate genes were selected if they were related to chlorophyll synthesis, stabilization, and decomposition and matched sequences in the NCBI database according to a BLASTx search. Of the 21 candidate genes potentially associated with the chlorophyll content (**Table 7**), *TraesCS1B02G066200*, *TraesCS5D02G559400*, *TraesCS5D02G559500*, *TraesCS5D02G559700*, *TraesCS6B02G126500*, and *TraesCS6B02G128000* encode chlorophyll protein/fat channels, while *TraesCS5D02G559400*, *TraesCS5D02G5G2*, and *TraesCS5G5G5G2* are widely associated with PII. There were six genes involved in chlorophyll biosynthesis (*TraesCS3B02G517200*, *TraesCS4B02G386100*, *TraesCS4B02G393600*, *TraesCS5D02G558200*, *TraesCS5D02G561700*, and *TraesCS6B02G527000*). Some of the genes were related to the regulation of chlorophyll motility, chlorophyll a/b-binding protein, and water channel stability, whereas other genes were associated with chlorophyll stability, including the chlorophyll kinase-encoding gene *TraesCS2B02G577700*, which affects chlorophyll breakdown.

**Table 7:** Candidate gene information.

Chr	Pos (MB)	Gene	Gene annotation or coding protein
1B	50.302948-50.303445	*TraesCS1B02G066200*	Protein FATTY ACID EXPORT 4, chloroplastic
2B	765.919066-765.920939	*TraesCS2B02G577700*	Phytol kinase 1
3B	760.334333-760.335358	*TraesCS3B02G517200*	F-box family protein
4B	664.77777850-664.780377	*TraesCS4B02G386100*	Protein WEAK CHLOROPLAST MOVEMENT UNDER BLUE LIGHT 1
4B	667.884245-667.885739	*TraesCS4B02G393600*	Heavy metal transport/detoxification superfamily protein
5D	560.186102-560.187499	*TraesCS5D02G558200*	Anthocyanin 5-aromatic acyltransferase
5D	560.872747-560.873199	*TraesCS5D02G559400*	Photosystem II CP47 reaction center protein
5D	560.873363-560.873479	*TraesCS5D02G559500*	Photosystem II reaction center protein T
5D	560.873763-560.873984	*TraesCS5D02G559700*	Photosystem II reaction center protein H
5D	562.028866-562.030167	*TraesCS5D02G561700*	Aquaporin
6B	121.820761-121.821111	*TraesCS6B02G126500*	Photosystem II reaction center protein H
6B	122.679445-122.682945	*TraesCS6B02G127000*	Heat Stress Transcription Factor family protein
6B	123.738176-123.746779	*TraesCS6B02G128000*	Zinc transporter, putative
6D	466.999116-466.999490	*TraesCS6D02G392400*	F-box domain containing protein, expressed
6D	469.310371-469.311897	*TraesCS6D02G397400*	Anthocyanin 3’-O-beta-glucosyltransferase
7A	197.300200-197.301901	*TraesCS7A02G227100*	Chlorophyll a-b binding protein, chloroplastic
7A	200.035948-200.039407	*TraesCS7A02G229700*	F-box family protein
7A	669.727531-669.729352	*TraesCS7A02G474200*	Peroxidase
7B	330.998271-330.998771	*TraesCS7B02G192800*	Acetyl-coenzyme A carboxylase carboxyl transferase subunit beta, chloroplastic
7B	339.208337-339.238733	*TraesCS7B02G196800*	envelope membrane protein, chloroplastic
7B	602.111303-602.112246	*TraesCS7B02G346300*	weak chloroplast movement under blue light protein (DUF827)

## 4 Discussion

### 4.1 Spectroscopic dynamics in wheat

Because chlorophyll is a major pigment in wheat, the reflectivity of light at various wavelengths is primarily influenced by the chlorophyll content of wheat. In this study on wheat, light reflectivity was high in all five major bands: near-infrared light > red edge light > green light > red light > blue light; the relatively low reflectivity of blue and red light was because chlorophyll predominantly absorbs both red and blue light (Zucchelli et al., 2012). When exposed to normal solar radiation, both red and blue light are absorbed by chlorophyll significantly more than other light (near-infrared and red and green edges). Thus, the rank order for reflectivity is as follows: near-infrared light > red edge light > green light > red light > blue light. After normal irrigation and drought treatments, the light reflectivity of each band gradually decreased as the duration of the reproductive period increased (i.e., from the Heading Stage to the Flowering Stage to the grain filling stage), reflecting gradual increases in the light absorption capacity of chlorophyll during wheat growth and development. These results were in accordance with chlorophyll content trends in growing wheat plants (i.e., Heading Stage< Flowering Stage< grain filling stage) (Jin-Peng et al., 2021).

In plants, energy is mainly derived from photosynthesis. The decreased light reflectivity in all five bands suggests that for wheat leaves, photosynthesis is associated with an increase in light absorption and efficiency. The energy capacity of wheat gradually increased during the heading, flowering, and grain filling stages. Red edge light refers to a region of the electromagnetic spectrum near the near-infrared band that induces rapid changes to vegetation reflectivity; it also intersects with red light (Horler et al., 1983) at wavelengths in the 670–760 nm range, in which light reflectivity increases substantially. Chlorophyll in wheat leaves absorbs most of the visible light, but the absorption of wavelengths longer than 700 nm is challenging. In wheat, single cell structures are the most important determinants of light reflectivity. The mechanism mediating reflection is similar to that of angular reflectors; the reflectivity of light at wavelengths between 680 and 730 nm can change rapidly from 5% to 50% (Filella and Penuelas, 1994). For this reason, wheat leaves were relatively bright and more reflective (in the infrared band) during the drone mission. For remote sensing, red edge light is often used for variables in the inversion model of the crop canopy index (Walsh et al., 2018).

In the present study, the leaf area index of wheat decreased and plant cover decreased under drought conditions. Hence, normal watering is necessary for heading and flowering under red edge light. Moreover, this explains why reflectivity was higher under normal irrigation conditions than under drought conditions. However, during the grain filling stage, reflectivity was higher under drought conditions than under normal irrigation conditions. Although drought stress can decrease the leaf area index and increase reflectivity, an increase in the chlorophyll content decreases red edge light reflectivity. In addition, red edge light reflectivity was lower under normal irrigation conditions than under drought conditions. At relatively high elevations, multispectral information reflects crop growth. Thus, drones may be used to accurately assess crop growth. At the same time, this approach enables scalable field phenotyping for screening drought-resilient wheat germplasm.

### 4.2 Expected and actual chlorophyll contents

The model used in this study, which was based on a BP neural network, indicated the predicted and measured values of the model were correlated (0.90–0.93; *R^2^* = 0.80–0.87) in the Heading Stage under normal irrigation conditions. Similarly, the predicted and measured values were also correlated in the Flowering Stage (0.91–0.92; *R^2^* = 0.83–0.84). The correlation between the predicted and measured values was also determined in the Grain Filling Stage (0.88–0.90; *R^2^* = 0.77–0.81). Following the drought stress treatment, the correlation between the predicted values obtained from the model and the measured values at the Heading Stage ranged from 0.57 to 0.70 (*R^2^* = 0.32–0.49). The correlations between predicted and measured values were 0.89–0.91 (*R^2^* = 0.79–0.83) and 0.94–0.96 (*R^2^* = 0.88–0.91) in the flowering and grain filling stages, respectively (**Table 4**). The correlation in the overall data reached 0.84, with a coefficient of determination of 0.71. The study findings suggest chlorophyll contents for two environments can be predicted using a model based on an artificial neural network.

Wei et al. (2020) used GOSVI, GNDVI, CARI, and other vegetation indices as well as stepwise regression to develop a set of SPAD inversion models for the Heading Stage of winter wheat; their *R^2^* values were as high as 0.81. The results of this study are presented in **Table 4**. During the Heading Stage, the *R^2^* value peaked at 0.87, which is higher than that in a conventional model. The correlation and *R^2^* values in this study are likely good because the centralized regression equation for the traditional method of modeling was abandoned and the method for building distributed models, which are weighted by each of the nodes, was used to predict the chlorophyll content. An advantage of this model is that, when distributed, the results are modulated by multiple highly precise calculations, which are accompanied by the accumulation of data in the database. Self-healing and model fitting can lead to increased accuracy (i.e., improvements via yearly fitting of new data). A disadvantage of this model is that it involves a complex calculation; this complexity will increase as data accumulates in the database. Because of the difficulty of manual calculations using a simple calculator, computational calculations are required for the inversion model developed to determine the chlorophyll content. In terms of predictions, the extreme values in two environments are influenced by the weighted nodes. This results in overly conservative data. Finally, the maximum predicted value was lower than the maximum measured value, whereas the minimum predicted value was higher than the minimum measured value.

The conservative nature of prediction results may be attributed to four main factors. First, the Backpropagation (BP) neural network utilizes mean squared error as the loss function and minimizes the error between predicted and actual values through gradient descent, which naturally causes predictions to gravitate towards the dataset’s mean. Second, during training, the BP neural network continuously adjusts weights and biases to minimize overall error, leading to a tendency to underestimate extreme values and overestimate minimal values, as extreme values occur less frequently in the training set, resulting in insufficient learning of these cases. Third, commonly used activation functions in BP networks (such as Sigmoid and tanh) exhibit compression properties, mapping inputs to a limited range and causing outputs to naturally bias towards central values rather than extremes. Finally, to prevent overfitting, BP networks often employ regularization techniques that constrain weight magnitudes, resulting in a tendency for more conservative predictions. While this enhances the model’s generalization ability, it can also lead to more cautious prediction outcomes.

The Heading Stage correlation coefficient was between 0.57 and 0.70 under drought stress conditions (*R^2^* = 0.32–0.49) at the two study sites. The *R^2^* value was lower than that in the other stages, but the predicted and measured values were significantly correlated. These results may be related to differences among the heading, flowering, and grain filling stages, among which the Heading Stage reportedly has the lowest leaf area index (Gao et al., 2021), which decreases further in response to drought stress. Considering drought stress or other factors can cause the leaf area index to decrease, the utility of the inversion model for extreme environments may need to be enhanced via a multi-year study involving the artificial neural network model. Natural populations were used as experimental materials in this study. Accordingly, the neural network model developed in the current study is likely versatile and useful for precisely measuring plant chlorophyll contents.

### 4.3 Correlations between predicted and measured chlorophyll contents

We used 36,873 SNP markers selected from a 50K chip as well as predicted chlorophyll contents of 119 experimental materials for a genome-wide association analysis conducted using the MLM Q + K model to control false positives due to the population structure and kinship. When a threshold of<0.001 was applied, 308 loci were identified across 21 chromosomes, explaining 7.58%–19.58% of the phenotypic variation (**Table 5**). This indicates that genes controlling the chlorophyll content are widely distributed throughout the genome. Moreover, the chlorophyll content is a quantitatively regulated trait influenced by multiple genes. Specifically, the association analysis of predicted chlorophyll values identified 206 loci (explaining 7.58%–19.58% of the phenotypic variation), while the association analysis of measured values identified 102 loci (explaining 9.31%–15.83% of the phenotypic variation). The broader phenotypic variation explained (%) for the 206 loci as-sociated with predicted values than for the 102 loci associated with measured values suggests predicted values may be useful for increasing loci coverage and number. This may be because the neural network model can establish a correlation between measured chlorophyll contents and spectral information for each material (Zhe et al., 2021), whereas manual measurements may introduce errors. Consequently, the model calibrates the measured data according to the relationship with spectral information, indicating that predicted values may more closely reflect the actual data than measured values.

Increases in the size of a neural network modeling ensemble (multi-year data modeling) are conducive to the calibration of the model. The number of loci identified for the predicted chlorophyll contents was greater than or equal to the number of loci detected for the measured chlorophyll contents (**Table 5**), indicating that the predicted loci were not highly enriched for false positives at certain times and under specific treatment conditions. **Table 5** presents details regarding the distribution of the 308 loci according to treatments and periods. On the basis of the predicted and measured chlorophyll contents, genes con-trolling chlorophyll levels were distributed across the entire genome. Thus, an inversion model based on a neural network can accurately predict chlorophyll contents. A correlation analysis of the actual and predicted chlorophyll content data detected 18 loci (ex-plaining 7.58%–19.58% of the phenotypic variation) on chromosomes 1A, 1B, 2B, 3B, 4B, 5A, 5B, 5D, 6B, 6D, 7A, and 7B (**Table 6**). There was considerable consistency between the actual and predicted chlorophyll contents, implying the data obtained from an improved model based on multispectral UAVs can gradually replace data obtained manually. Chlorophyll measured value-based loci (P = 9.91 × 10^−5^–9.90 × 10^−4^; mean of 6.71 × 10^−4^) explained 9.31%–15.83% of the phenotypic variation (mean of 10.82%). For the predicted chlorophyll contents, the P-value was 5.77 × 10^−6^–1.00 × 10^−3^ (mean of 4.25 × 10^−4^). The phenotypic variation explained varied between 7.58% and 19.58% (mean of 11.50%). Correlations be-tween the loci detected according to measured and predicted data were analyzed. The mean P-value of the predicted chlorophyll content-related loci was low, reflecting a strong correlation between predicted values and the loci identified according to predicted values (Pal et al., 2021). The loci detected using predicted chlorophyll contents also accounted for a relatively large proportion of the phenotypic variation (on average). Hence, in terms of quality, the loci identified according to predicted chlorophyll contents were likely better than the loci detected on the basis of measured chlorophyll contents.

Predicted chlorophyll contents were analyzed and compared with the measured values. Compared with the loci identified using measured values, the loci identified using predicted values were more abundant, had stronger associations, and explained more of the phenotypic variation. Multi-year data may improve the output of the neural network model, but they may also be used instead of artificially determined chlorophyll contents.

### 4.4 Prediction of functional genes associated with the chlorophyll content

Genome-wide association analysis of predicted and measured values revealed 308 loci significantly associated with the chlorophyll content. These loci were used to search the Chinese Spring Genome Database to detect similar sequences in common wheat. Twenty-one candidate genes associated with the chlorophyll content (i.e., related to chlorophyll synthesis, stabilization, or decomposition) were identified following a BLASTx-based screening of the NCBI database (**Table 7**). Notably, *TraesCS1B02G066200*, *TraesCS5D02G559400*, *TraesCS5D02G559500*, *TraesCS5D02G559700*, *TraesCS6B02G126500*, *TraesCS6B02G128000* (encoding chlorophyll protein/fat channels), *TraesCS5D02G559400*, *TraesCS5D02G5G2*, and *TraesCS5G5G5G2* (related to PII) were among the candidate genes. Furthermore, six genes are involved in chlorophyll biosynthesis, including the gene encoding a photosystem II reaction center protein associated with the chlorophyll a/b-binding protein complex (Shen et al., 2021). The protein–lipid pathway regulates chlorophyll synthesis in chloroplasts (Wang, 2020). Zinc transport in plants is controlled by zinc transporters; it is possible that either too little or too much zinc may affect chlorophyll synthesis (Zhang et al., 2020). The following genes are associated with chlorophyll stability: *TraesCS3B02G517200*, *TraesCS4B02G386100*, *TraesCS4B02G393600*, *TraesCS5D02G558200*, *TraesCS5D02G561700*, and *TraesCS6B02G527000*. Other genes are related to chlorophyll motility, chlorophyll a/b-binding protein, water channel stability, and chlorophyll stability. In previous studies, F box (Guérin et al., 2021) and anthocyanin (Sharma et al., 2020) were revealed to increase plant strength and maintain normal physiological and biochemical activities during exposures to stress, while also stabilizing chlorophyll. Chloroplast motility can be regulated by blue light as an adaptive response to environmental changes (Qiao et al., 2014). Water channel proteins regulate the ingress and egress of water molecules, thereby maintaining water levels in plants exposed to drought stress (Li et al., 2022). *TraesCS2B02G577700* encodes a chlorophyll kinase associated with chlorophyll degradation. The formation of branched chains of chlorophylls influences the solubility of chlorophyll lipids. The phosphorylation of chlorophyll by kinases adversely affects stability, ultimately leading to chlorophyll degradation (Philipp et al., 2021). The gene TraesCS7A02G474200, which encodes peroxidase, is associated with lead tolerance, indicating its role in responding to abiotic stress (Zhi et al., 2022). Phytol kinase 1 may be involved in regulating drought resistance in plants Zhang (2023). Aquaporin in alfalfa (Medicago sativa) might participate in the regulation of drought tolerance (Jiang et al., 2025).

### 4.5 GO and KEGG enrichment analyses of candidate genes

**Figure 7:**
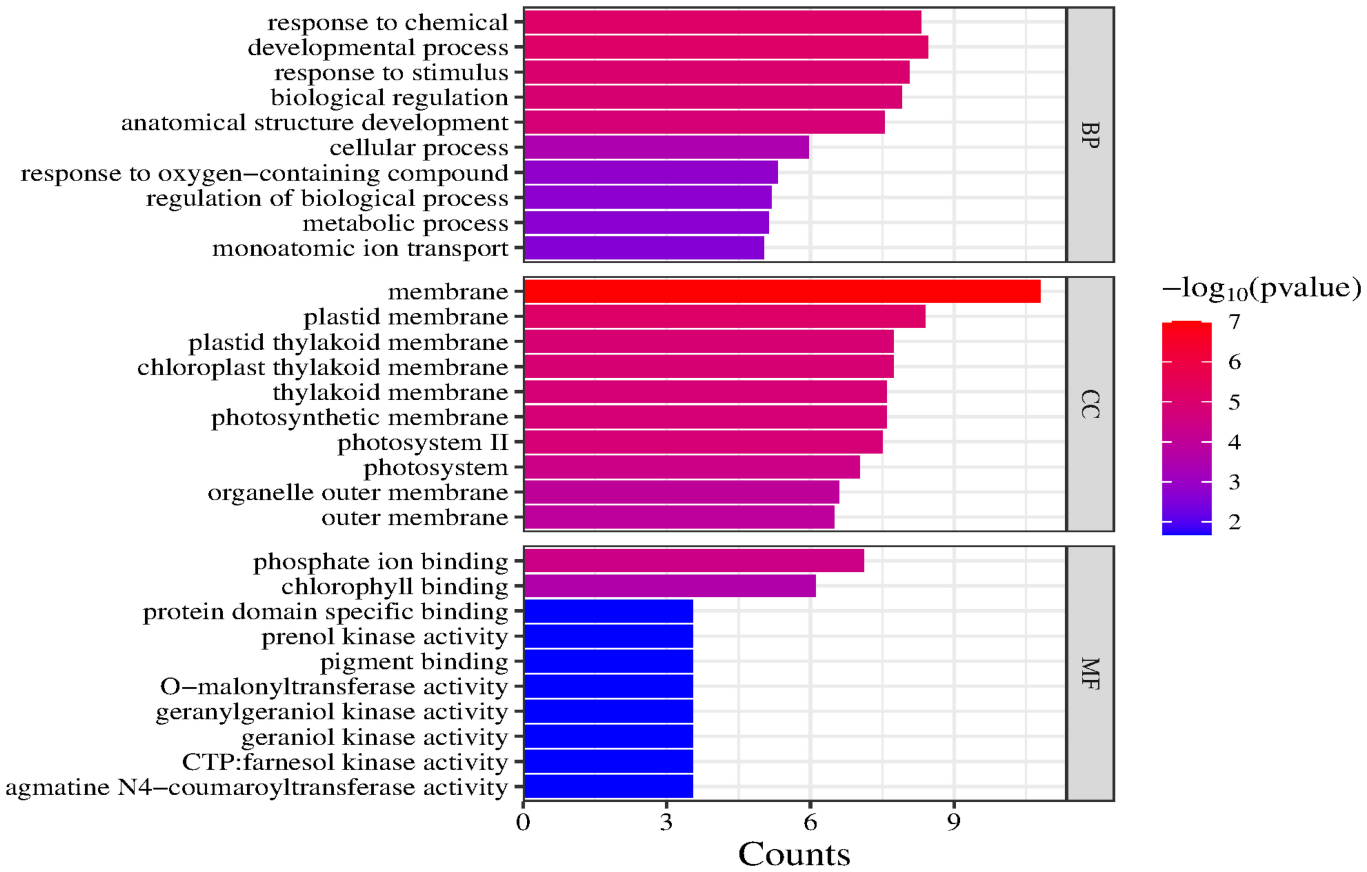
GO enrichment analysis of candidate genes (top 30).

GO and KEGG enrichment analyses were performed using 21 candidate genes. The GO analysis revealed that the candidate genes were primarily enriched in two biological processes (developmental process and response to chemicals), indicating that they may con-tribute to various environmental responses and developmental activities. In terms of cellular components, the candidate genes were significantly associated with membranes and plastid membranes, suggesting that they encode proteins primarily localized to cell membranes and related structures. The main molecular functions associated with the candidate genes were phosphate ion binding and chlorophyll binding, highlighting their importance for photosynthesis and metabolic regulation (**Figure 7**).

**Figure 8:**
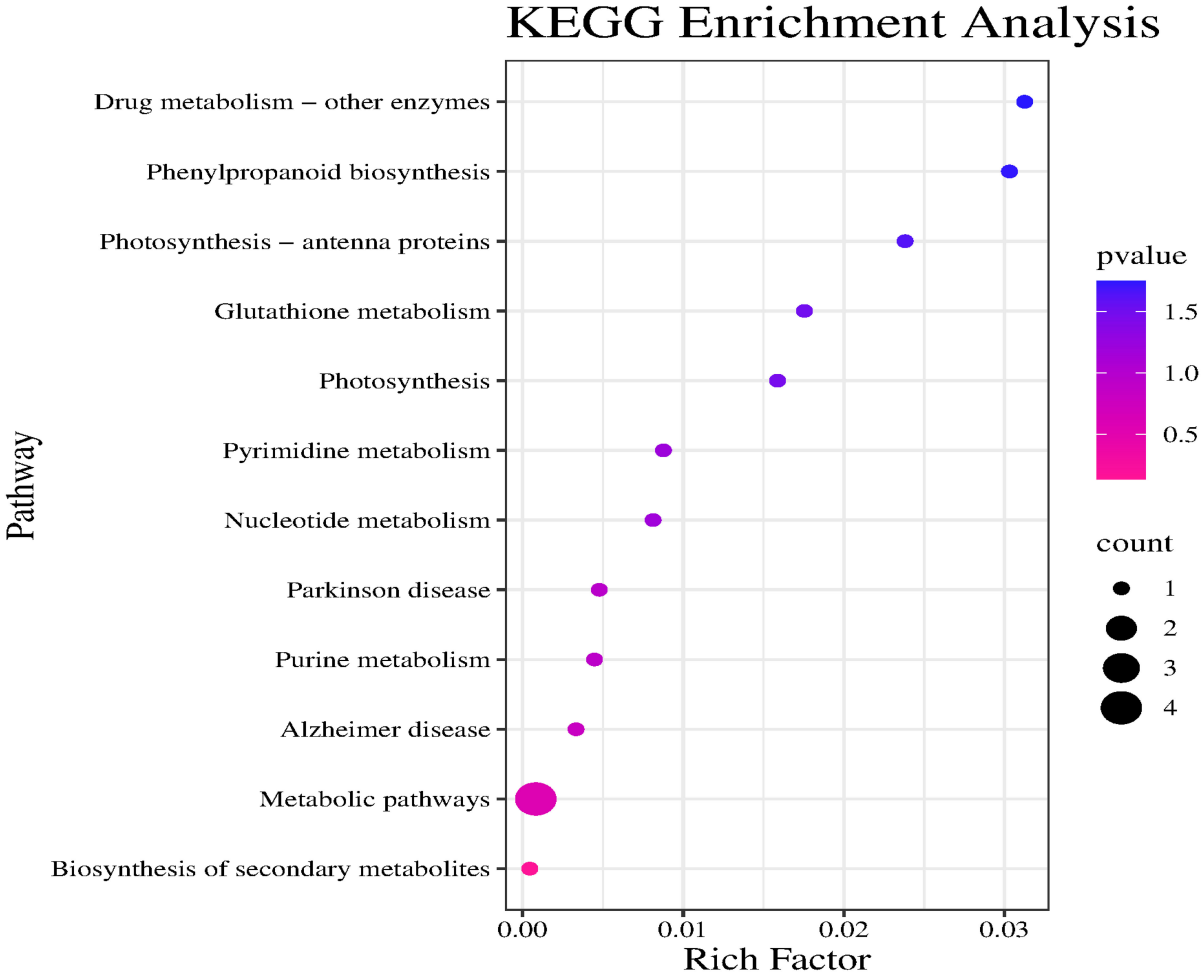
KEGG enrichment analysis of candidate gene.

The major enriched KEGG pathways among these candidate genes were Drug metabolism – other enzymes, Phenylpropanoid biosynthesis, Photosynthesis – antenna proteins, and Metabolic pathways. Notably, Drug metabolism – other enzymes was the most significantly enriched pathway. Although the Metabolic pathways category included the most target genes, its significance was relatively low. Drug metabolism – other enzymes, Phenylpropanoid biosynthesis, and Photosynthesis – antenna proteins had high enrichment factors and significance, suggestive of a relatively high proportion of target genes; however, there were relatively few specific genes associated with these pathways (**Figure 8**).

### 4.6 Novelty of the study and its implications for wheat improvement

The innovation of this study lies in the combination of multispectral unmanned aerial vehicle (UAV) technology with manual measurements, achieving efficient and accurate prediction of chlorophyll content, thus providing a new methodology for wheat gene mining. By constructing an inversion model based on remote sensing data, we were able to assess the dynamic changes of chlorophyll content at different growth stages (heading, flowering, and grain filling). This method not only increases the throughput of phenotypic analysis but also offers a new perspective on the association between genotype and phenotype, thereby advancing the process of wheat improvement.

The results indicate that changes in chlorophyll content are closely related to the growth stages of wheat under both normal and drought conditions. Drought and irrigation significantly impact the process of chlorophyll content prediction. Specifically, under drought conditions, the prediction accuracy of chlorophyll content is relatively lower, but the correlation in the flowering and grain filling stages is significantly enhanced, suggesting that changes in chlorophyll may be closely related to the physiological adaptation mechanisms of wheat in response to water stress. Therefore, understanding the effects of different environmental conditions (such as drought and irrigation) on chlorophyll content and its prediction can provide important theoretical foundations for wheat improvement.

Moreover, this study identified multiple loci associated with chlorophyll content through genome-wide association analysis, which exhibited varying phenotypic variations under different stress conditions. This finding indicates the potential to identify genomic regions through marker-trait association analysis, providing a scientific basis for the genetic improvement of wheat. Future research should further explore how these genetic markers can be applied in breeding practices to achieve more efficient wheat improvement strategies.

In summary, this study not only offers a new tool for rapid assessment of chlorophyll phenotypes in wheat but also deepens the understanding of wheat’s physiological responses under different growth conditions at the genomic level, providing important theoretical support for enhancing wheat resilience.

## 5 Conclusions

Chlorophyll contents can be accurately reflected by spectral information. A neural network model and spectral information are useful for an inversion model-based determination of the chlorophyll content of plant samples. In this study, a correlation analysis involving measured and predicted chlorophyll contents showed that the correlation between the measured and predicted chlorophyll contents was relatively high (correlation coefficient reaching 0.87). Furthermore, using predicted chlorophyll contents may be conducive to increasing the number and quality of identified genomic loci related to the chlorophyll content. Therefore, the study model may be applied to rapidly determine chlorophyll contents, thereby enriching relevant databases.

## Data Availability

The original contributions presented in the study are included in the article/supplementary material. Further inquiries can be directed to the corresponding author.
