# Supplementary material for: Genome-wide association study of wheat chlorophyll dynamics under drought and irrigation using multispectral UAV phenotyping
Source: Front Plant Sci. 2025 Sep 4;16:1607862. doi: 10.3389/fpls.2025.1607862 (PMC12443573; doi:10.3389/fpls.2025.1607862)
Supplement: Supplementary file 1 [file Table1.docx]

| Schedule 1Distribution table of 119 material subgroups | | | |
| --- | --- | --- | --- |
| serial number | Breed name | source | subgroups |
| 1 | Abogeqilige | landraces | 1 |
| 2 | Akebudayi | landraces | 1 |
| 3 | Akekuzigai | landraces | 1 |
| 4 | White winter wheat | landraces | 1 |
| 5 | Haaretuosiman | landraces | 1 |
| 6 | Hexi Buck red wheat | landraces | 1 |
| 7 | Kayimuhong | landraces | 1 |
| 8 | Kezibudayi | landraces | 1 |
| 9 | Kuqa white winter wheat | landraces | 1 |
| 10 | Lawadelang | landraces | 1 |
| 11 | Mailuowaxi | landraces | 1 |
| 12 | Nawatiran | landraces | 1 |
| 13 | Qilike | landraces | 1 |
| 14 | Suilai small red winter wheat | landraces | 1 |
| 15 | Small white winter wheat | landraces | 1 |
| 16 | Chang Bashman | landraces | 1 |
| 17 | Han 5316 | Introduced varieties (lines) | 2 |
| 18 | Jimai 24 | Introduced varieties (lines) | 2 |
| 19 | Jimai 26 | Introduced varieties (lines) | 2 |
| 20 | Tangshan 6898 | Introduced varieties (lines) | 2 |
| 21 | F49-70 | Introduced varieties (lines) | 2 |
| 22 | Afulee | Introduced varieties (lines) | 2 |
| 23 | Odessa-16 | Introduced varieties (lines) | 2 |
| 24 | Odessa 3 | Introduced varieties (lines) | 2 |
| 25 | Baiqubao | Introduced varieties (lines) | 2 |
| 26 | Beijing 6 | Introduced varieties (lines) | 2 |
| 27 | Beijing 7 | Introduced varieties (lines) | 2 |
| 28 | Beijing 8 | Introduced varieties (lines) | 2 |
| 29 | Bima 1 | Introduced varieties (lines) | 2 |
| 30 | Bima 4 | Introduced varieties (lines) | 2 |
| 31 | Bima 6 | Introduced varieties (lines) | 2 |
| 32 | Caucasus | Introduced varieties (lines) | 2 |
| 33 | Gongnong19 | Introduced varieties (lines) | 2 |
| 34 | hongqi 1 | Introduced varieties (lines) | 2 |
| 35 | Huabei 187 | Introduced varieties (lines) | 2 |
| 36 | Huabei 497 | Introduced varieties (lines) | 2 |
| 37 | Jinan 4 | Introduced varieties (lines) | 2 |
| 38 | Luofulin 10 | Introduced varieties (lines) | 2 |
| 39 | Luofulin 13 | Introduced varieties (lines) | 2 |
| 40 | Mironov Memorial Mai 50 | Introduced varieties (lines) | 2 |

Continued

| 41 | Nongda 183 | Introduced varieties (lines) | 2 |
| --- | --- | --- | --- |
| 42 | Shanqian 2 | Introduced varieties (lines) | 2 |
| 43 | Wumang 1 | Introduced varieties (lines) | 2 |
| 44 | Wumang 4 | Introduced varieties (lines) | 2 |
| 45 | Xiaoe 186 | Introduced varieties (lines) | 2 |
| 46 | Xiaoheimai 46/131 | Introduced varieties (lines) | 2 |
| 47 | New Ukraine 83 | Introduced varieties (lines) | 2 |
| 48 | New Ukraine 84 | Introduced varieties (lines) | 2 |
| 49 | Youpilaina 1 | Introduced varieties (lines) | 2 |
| 50 | Youbao | Introduced varieties (lines) | 2 |
| 51 | Zaoyangmai | Introduced varieties (lines) | 2 |
| 52 | Kuidong 4 | self-bred varieties (lines) | 3 |
| 53 | Kuihua 2 | self-bred varieties (lines) | 3 |
| 54 | Shidong 7 | self-bred varieties (lines) | 3 |
| 55 | Shidong 9 | self-bred varieties (lines) | 3 |
| 56 | Xingdong 14 | self-bred varieties (lines) | 3 |
| 57 | Xingdong 15 | self-bred varieties (lines) | 3 |
| 58 | Xingdong 16 | self-bred varieties (lines) | 3 |
| 59 | Xingdong 22 | self-bred varieties (lines) | 3 |
| 60 | Xingdong 33 | self-bred varieties (lines) | 3 |
| 61 | Yinong 20 | self-bred varieties (lines) | 3 |
| 62 | Changdong 5 | self-bred varieties (lines) | 3 |
| 63 | Jiudong 1 | self-bred varieties (lines) | 3 |
| 64 | Jiudong 2 | self-bred varieties (lines) | 3 |
| 65 | Kadong 1 | self-bred varieties (lines) | 3 |
| 66 | Kadong 4 | self-bred varieties (lines) | 3 |
| 67 | Xingdong 2 | self-bred varieties (lines) | 3 |
| 68 | Xingdong 5 | self-bred varieties (lines) | 3 |
| 69 | Xingdong 7 | self-bred varieties (lines) | 3 |
| 70 | XC35 | self-bred varieties (lines) | 3 |
| 71 | XC37 | self-bred varieties (lines) | 3 |
| 72 | XC38 | self-bred varieties (lines) | 3 |
| 73 | XC39 | self-bred varieties (lines) | 3 |
| 74 | Shidong 8 | self-bred varieties (lines) | 3 |
| 75 | Xingdong 18 | self-bred varieties (lines) | 3 |
| 76 | Xingdong 19 | self-bred varieties (lines) | 3 |
| 77 | Xingdong 20 | self-bred varieties (lines) | 3 |
| 78 | Xingdong 21 | self-bred varieties (lines) | 3 |
| 79 | Xingdong 23 | self-bred varieties (lines) | 3 |
| 80 | Xingdong 24 | self-bred varieties (lines) | 3 |
| 81 | Xingdong 27 | self-bred varieties (lines) | 3 |
| 82 | Xingdong 28 | self-bred varieties (lines) | 3 |

Continued

| 83 | Xingdong 29 | self-bred varieties (lines) | 3 |
| --- | --- | --- | --- |
| 84 | Xingdong 31 | self-bred varieties (lines) | 3 |
| 85 | Xingdong 34 | self-bred varieties (lines) | 3 |
| 86 | Xingdong 37(CA9719) | self-bred varieties (lines) | 3 |
| 87 | Xingdong 41:01271 | self-bred varieties (lines) | 3 |
| 88 | Yinong 21 | self-bred varieties (lines) | 3 |
| 89 | XC02 | self-bred varieties (lines) | 3 |
| 90 | XC21 | self-bred varieties (lines) | 3 |
| 91 | XC26 | self-bred varieties (lines) | 3 |
| 92 | XC30 | self-bred varieties (lines) | 3 |
| 93 | XC31 | self-bred varieties (lines) | 3 |
| 94 | XC33 | self-bred varieties (lines) | 3 |
| 95 | XC34 | self-bred varieties (lines) | 3 |
| 96 | Chinese Spring | self-bred varieties (lines) | 3 |
| 97 | Hongzhitou/10 | self-bred varieties (lines) | 3 |
| 98 | Kuihua 1 | self-bred varieties (lines) | 3 |
| 99 | Xingdong 17 | self-bred varieties (lines) | 3 |
| 100 | Xingdong 30 | self-bred varieties (lines) | 3 |
| 101 | Xingdong 32 | self-bred varieties (lines) | 3 |
| 102 | Xingdong 36 | self-bred varieties (lines) | 3 |
| 103 | Yinong 16 | self-bred varieties (lines) | 3 |
| 104 | Yinong 18 | self-bred varieties (lines) | 3 |
| 105 | Yinong 19 | self-bred varieties (lines) | 3 |
| 106 | XC03 | self-bred varieties (lines) | 3 |
| 107 | XC06 | self-bred varieties (lines) | 3 |
| 108 | XC07 | self-bred varieties (lines) | 3 |
| 109 | XC11 | self-bred varieties (lines) | 3 |
| 110 | XC14 | self-bred varieties (lines) | 3 |
| 111 | XC17 | self-bred varieties (lines) | 3 |
| 112 | XC20 | self-bred varieties (lines) | 3 |
| 113 | XC22 | self-bred varieties (lines) | 3 |
| 114 | XC23 | self-bred varieties (lines) | 3 |
| 115 | XC27 | self-bred varieties (lines) | 3 |
| 116 | XC28 | self-bred varieties (lines) | 3 |
| 117 | XC29 | self-bred varieties (lines) | 3 |
| 118 | XC40 | self-bred varieties (lines) | 3 |
| 119 | Xinhan 688 | self-bred varieties (lines) | 3 |
